# A novel LUAD prognosis prediction model based on immune checkpoint-related lncRNAs

**DOI:** 10.3389/fgene.2022.1016449

**Published:** 2022-09-21

**Authors:** Yang Liu, Mingyang Yu, Xuechao Cheng, Xingshu Zhang, Qian Luo, Sijin Liao, Zhongzheng Chen, Jianhao Zheng, Kaijun Long, Xingwei Wu, Wendong Qu, Ming Gong, Yongxiang Song

**Affiliations:** Department of Thoracic Surgery, The Affiliated Hospital of Zunyi Medical University, Zunyi, Guizhou, China

**Keywords:** lung adenocarcinoma, lncRNA, immune check point, tumor microenvironment, bioinformatic analyse

## Abstract

Lung adenocarcinoma (LUAD) is a malignant disease with an extremely poor prognosis, and there is currently a lack of clinical methods for early diagnosis and precise treatment and management. With the deepening of tumor research, more and more attention has been paid to the role of immune checkpoints (ICP) and long non-coding RNAs (lncRNAs) regulation in tumor development. Therefore, this study downloaded LUAD patient data from the TCGA database, and finally screened 14 key ICP-related lncRNAs based on ICP-related genes using univariate/multivariate COX regression analysis and LASSO regression analysis to construct a risk prediction model and corresponding nomogram. After multi-dimensional testing of the model, the model showed good prognostic prediction ability. In addition, to further elucidate how ICP plays a role in LUAD, we jointly analyzed the immune microenvironmental changes in LAUD patients and performed a functional enrichment analysis. Furthermore, to enhance the clinical significance of this study, we performed a sensitivity analysis of common antitumor drugs. All the above works aim to point to new directions for the treatment of LUAD.

## Introduction

Lung cancer, as one of the most common types of cancer all over the world, has gained much attention in recent years (Cao et al., 2020; Ferlay et al., 2018). It was estimated that 2.09 million new cases were newly diagnosed, and 1.76 million patients died in 2018 (Bray et al., 2018). According to histological types, lung cancer could be classified as non-small cell lung cancer (NSCLC) and small cell lung cancer (SCLC) roughly, and lung adenocarcinoma (LUAD) was the major subtype that accounted for over one million worldwide deaths annually (Zhang et al., 2020a). Smoking has become the most common risk factor for LUAD (Gould et al., 2007). Several approaches have been used in the clinical treatment of LUAD patients, mainly including radiotherapy, chemotherapy, and surgical resection according to the TNM system (Nasim et al., 2019). When progressed to advanced stages, survival decreased monthly sharply, so it is of great need for early diagnosis and intervention (Steven et al., 2016). Along with the rapid growth of large-scale genomic studies in recent decades, some somatic mutations associated with LUAD have been noticed like TP53, KRAS, EGFR, et al., which emphasized the importance of immunotherapies (Campbell et al., 2016). Meanwhile, for advanced LUAD, the effect of chemotherapy was greatly limited by its malignant nature, and immunotherapy seed to be the most effective approach to provide early diagnosis and improve survival status (Zheng et al., 2021). So, more immune therapeutic targets are needed for better and more precise clinical diagnosis and prognosis.

With the growing development in immunotherapy, several types with different mechanisms of action have been applied in clinical treatment, like vaccinations, monoclonal antibodies, and checkpoint inhibitors (Abbott and Ustoyev, 2019). Oncolytic vaccines were created in the 1920s and shelved until 1976 due to lake of understanding of the specific mechanism.

As an effective method for non-Hodgkin’s lymphoma, rituximab has gradually been used in many types of cancer as an important monoclonal antibody (Ribatti, 2014). The latest immune checkpoint (ICP) proteins, like programmed cell death protein 1 (PD-1) and antibodies against cytotoxic T lymphocyte antigen-4 (CTLA-4) also have been fully investigated (Thompson, 2018). The former is a cell-surface receptor expressed on immune cell types, while the latter mainly reduces interleukin 2 (IL-2) production and T-cell proliferation (Kennedy and Salama, 2020). As for a novel T cell-target method, chimeric antigen receptor (CAR) T cell therapies have been developed and approved for clinical use mainly in hematological cancers owing to the delivery barriers faced by solid tumors (Fesnak et al., 2016). Therefore, it is of great importance to explore novel targets for solid tumors, especially LUAD.

Long non-coding RNAs (lncRNAs) represent a major class of regulatory non-coding RNAs larger than 200 nt in length (Peng et al., 2017). Altered immune infiltration is a hallmark of the tumor, and it is well recognized that lncRNAs regulate the immune response in cancer progression (Zhang et al., 2020b). Some studies demonstrated that the ectopic expression of lncRNA-cell division cycle six promoted proliferation and metastasis of breast cancer cells *via* regulation of the G1 phase checkpoint, demonstrating a critical effect in tumor development (Kong et al., 2019). Meanwhile, much emphasis has been put on the tumor microenvironment (TME) to further elucidate the immune alteration which influences tumor development apart from tumor cells. In solid tumors, TME consists of several types of immune cells and stromal cells, like cancer-associated fibroblasts (CAFs), regulatory T cells (Tregs), myeloid-derived macrophages (MDSCs), etc. (Mu and Najafi, 2021). While the correlation between lncRNAs and TME remains a mystery.

Thus, we conducted an overall immune checkpoint-related lncRNAs risk and prognostic model in patients with LUAD, trying to explore risk factors for cancer clinical care through bioinformatics technique and survival analysis, and provide potential therapeutic targets for clinical treatment.

## Materials and methods

### Data acquisition and processing

All relevant LUAD patients’ information and data in this study were downloaded from the TCGA database (Blum et al., 2018). After excluding samples with missing prognostic information or survival time of fewer than 30 days, finally, 490 LUAD samples were included in this study. These samples are randomly divided into the training set and testing set. A total of 246 samples in the training set were used to develop a predictive risk model. The testing set included 244 samples used to validate the established risk model. The 47 ICP genes were derived from the latest research results of Liu et al. (2022) (Supplementary Table S1). ICP-related lncRNAs were obtained by Pearson’s correlation test (Pearson correlation coefficient >0.4, *p* < 0.001), and 2,061 ICP-related lncRNAs were identified.

### Differential RNA screening

The expression levels of lncRNAs and mRNA were extracted from the transcriptome data of LUAD and normal samples, respectively, and lncRNA expression and differential analysis were performed using the “limma” package, where genes with FDR < 0.05 and |logFC| > 1 were considered to have significant differences.

### Construction of risk models

Combined with the prognostic information of patients, univariate regression analysis was used to screen the differential ICP-related lncRNAs associated with prognosis. Afterward, we used LASSO regression (R package “glmnet”, version 4.1-3) to run 1,000 cycles of 10-fold cross-validation with *p* < 0.05. Finally, through multivariate regression analysis, a 14 ICP-related lncRNAs risk model was constructed.

We calculated the risk score with the following formula:

$$Risk\;score=\sum_{k=1}^{n}Coef(\ln cRNA)*expr\left(\ln cRNA^{k}\right)$$

where *Coef* (ln*cRNA*) represents the correlation coefficient between lncRNAs and survival, and *expr* (ln*cRNA* ^*K*^) represents the expression of lncRNAs. All selected LUAD samples were divided into high-risk and low-risk groups based on the mean risk scores.

### Risk model testing and evaluation

Through univariate/multivariate regression analysis, ROC curves were performed (“glment,” “survminer,” and “survival” R packages) to test whether the risk model could be used as an independent predictor of prognosis in LUAD patients. In total, we calculated and plotted 1-, 3-, and 5-years ROC curves.

### Survival analysis and principal component analysis

Kaplan–Meier (K-M) survival analysis was used to determine the overall survival (OS) of LUAD patients between two groups by the “survival” package. Principal component analysis (PCA) is used for efficient dimensionality reduction, model identification, and group visualization of high-dimensional data.

### Nomogram construction

To better guide the clinical diagnosis and treatment of LUAD, we combined the risk scores, and other clinical features to construct a nomogram by “rms” package.

### Tumor microenvironment and immunotherapy analysis

Using the “maftools” R package, tumor mutational burdens (TMBs) in LUAD patients were assessed. Furthermore, the CIBERSORT (Newman et al., 2015) and ssGSEA algorithm, as well as TIMER (http://timer.comp-genomics.org) {Li, 2017 #21} were performed to evaluate the immune cell infiltration status in different risk groups. To predict the efficacy of clinical immunotherapy in LUAD patients, we used Tumor Immune Dysfunction and Exclusion (TIDE) prediction.

### Drug sensitivity analysis

IC50 of each LUAD patient relative to a common antineoplastic drug was determined as the patient’s sensitivity to this drug using the Genomics of Drug Sensitivity in Cancer (GDSC) platform (Yang et al., 2013) and used R package pRRophetic (version 0.5) for calculation and visualization.

### Functional analysis

GSEA analysis was done using gene set enrichment analyses software (https://www.gsea-msigdb.org/gsea/login.jsp) (Subramanian et al., 2005). GO and KEGG enrichment analyses based on the differential genes between high and low-risk groups were performed using DAVID online site (version 6.8), where relevant annotations with *p* < 0.05 and FDR < 0.05 were considered significantly different. Additionally, competitive endogenous RNA (ceRNA) networks were constructed and visualized using Cytoscape (version 3.6.1).

### Statistical analysis

All statistical analyses were performed in R software (version 4.1.1). Differences between groups were compared using the Wilcoxon rank-sum test. K-W tests were used to compare differences between three or more groups. Statistical significance was defined as a *p* < 0.05 if the above methods were not specifically stated.

## Results

### Expression and copy number variation of immune checkpoints-related genes in LUAD

**FIGURE 1:**
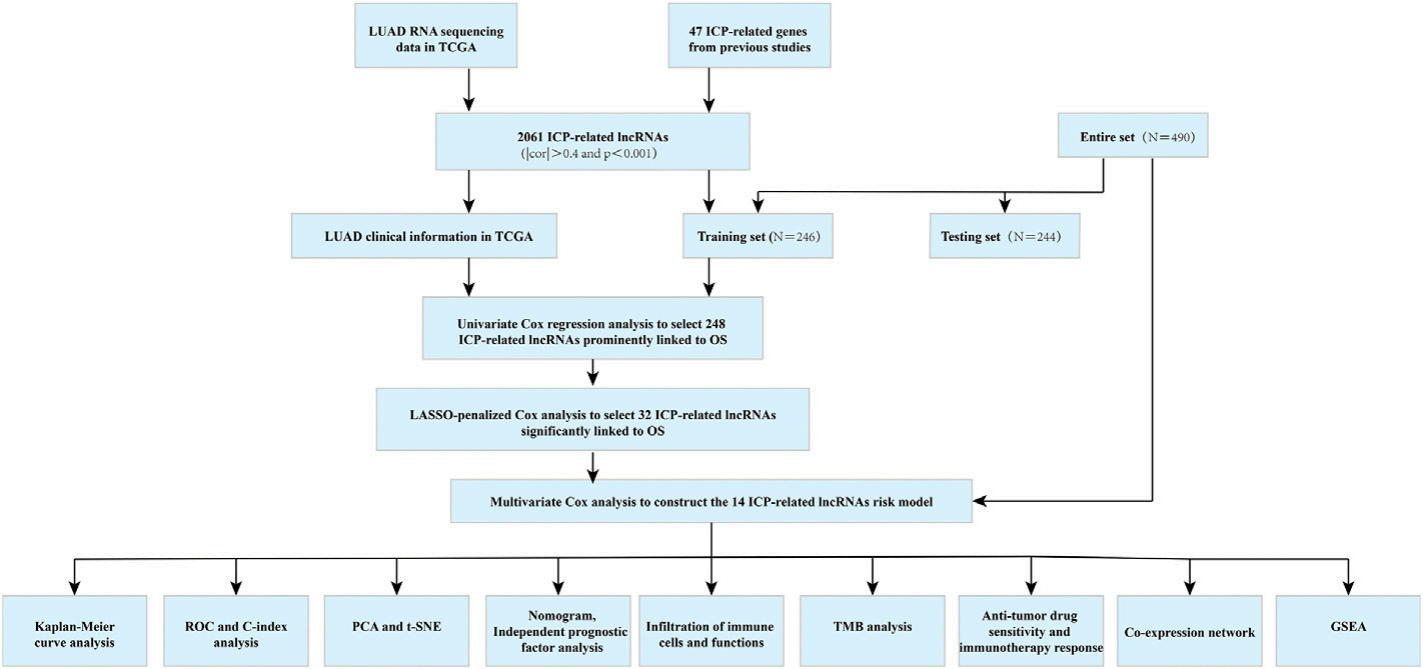
Workflow chart.

**FIGURE 2:**
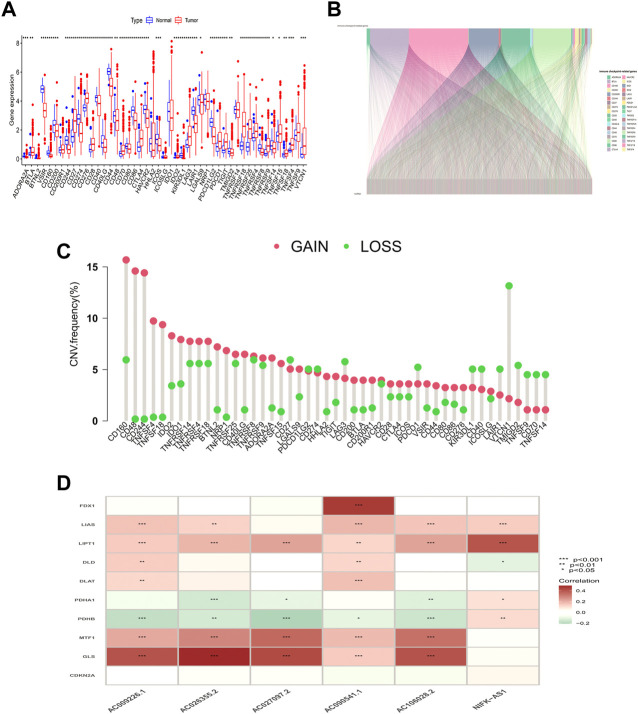
Genetic and expression variation of the MRDEGs in LUAD patients. **(A)** ICP-related gene expression profile. **(B)** Sankey relation diagram for ICP-related genes and lncRNAs. **(C)** The CNV frequency of 47 ICP genes in the LUAD cohort. **(D)** Heatmap for the correlations between randomly ICP-related genes and lncRNAs.

The workflow is shown in Figure 1. Forty-seven ICP-related genes were obtained for further analysis (Supplementary Table S1) as well as LUAD patients’ clinical features can be found in Supplementary Table S2. Differences in the expression of ICP-related genes between 535 tumor tissues and 59 normal tissue samples are shown in Figure 2A. Additionally, somatic copy number variation (CNV) among 47 ICP genes was studied (Figure 2C). The ICP-related lncRNAs interaction network is shown in the form of the Sankey diagram (Figure 2B, r > 0.4, *p* < 0.001), and the correlation between ICP-related genes and lncRNAs is shown in Figure 2D.

### Risk model construction and validation

**FIGURE 3:**
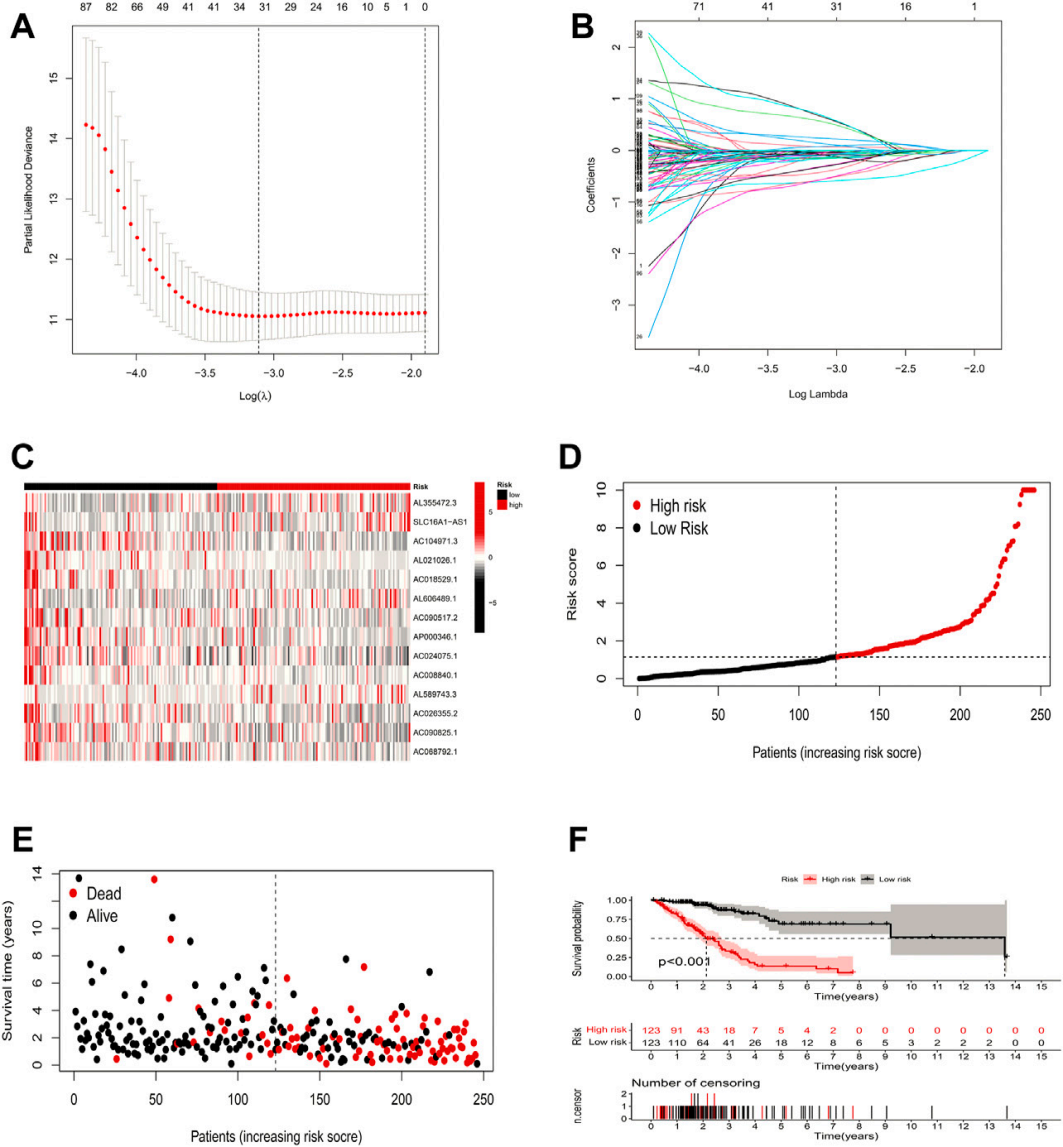
Risk model construction and validation. **(A,B)** Result of LASSO regression analysis. **(C)** Heatmap to show the expression of 14 lncRNAs between high- and low-risk groups in the training set. **(D)** Expression differences of 14 ICP-related lncRNAs in different risk groups in the training set. **(E)** Distribution of sample risk score and different patterns of survival status/time between the high-risk and low-risk groups in the training set. **(F)** Kaplan-Meier curve of high-risk and low-risk patients in the training set.

In this study, 248 ICP-related lncRNAs were screened by using univariate Cox regression analysis (Supplementary Table S3). To prevent overfitting prognostic features, we further performed LASSO Cox analysis and 32 lncRNAs that were highly correlated with LUAD prognosis **(** Figures 3A,B). Finally, 14 ICP-related lncRNAs with the strongest prognostic predictive ability were identified by multivariate COX regression analysis (Supplementary Table S4) for risk model construction.

The formula for the risk score is:

Risk scores = AL355472.3∗(1.7297898132188) + SLC16A1-AS1∗ (2.02045359749171) + AC104971.3∗(−0.861884680221223) + AL021026.1∗(−1.92527800701289) + AC018529.1∗(−1.41451695188048) + AL606489.1∗(0.484465069923853) + AC090517.2∗(−0.67755983967278) + AP000346.1∗(−1.34031276829931) + AC024075.1∗(−0.456581688150894) + AC008840.1∗(−1.67186147401095) + AL589743.3∗(1.01756516292608) + AC026355.2∗(−0.383441820151563) + AC090825.1∗(−0.894102424172019) + AC068792.1∗(−0.778661427765015).

With the above signatures, the patient’s prognostic risk score was calculated. For each patient, the relative expression levels of 14 ICP-related lncRNAs are presented in Figure 3C. Based on the mean risk scores, we divided all LUAD patient samples into high-risk and low-risk groups, where the patient distribution in the high-risk and low-risk groups of the training set is shown in Figure 3D. Figure 3E demonstrates the survival status and survival time of patients in the high-risk and low-risk groups in the training set. Figure 3F shows the prognosis and survival of patients in different risk groups in the training set (based on K-M survival analysis). It can be seen that the prognosis of patients can be clearly distinguished in the training set after changing the risk model (*p* < 0.001).

**FIGURE 4:**
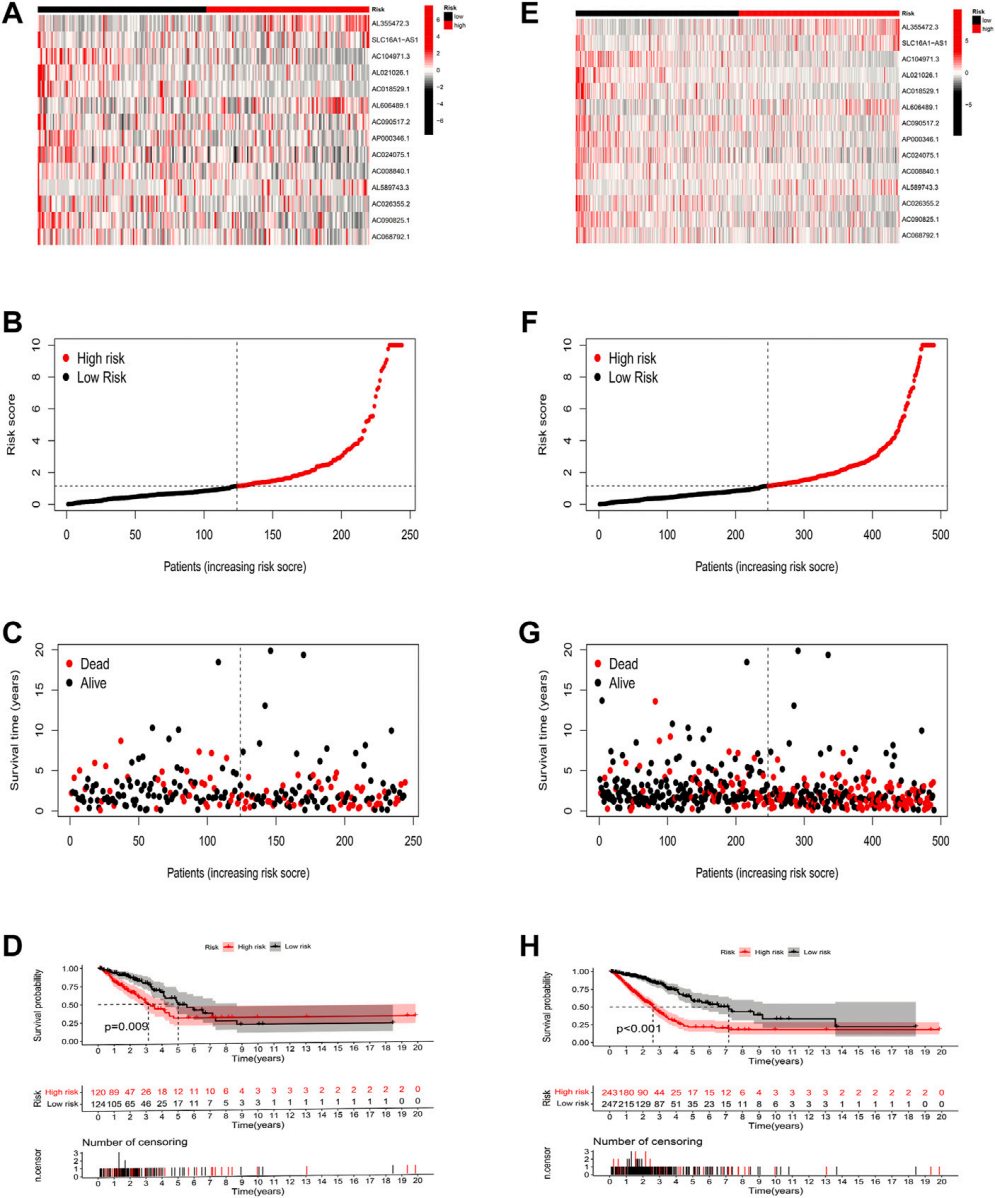
Risk model construction and validation in testing and entire sets. **(A–D)** The expression of 14 key prognostic lncRNAs in the testing set, the survival status of LUAD patients, the risk score, and the results of K-M analysis of survival analysis. **(E–H)** The expression of 14 key prognostic lncRNAs in the entire set, the survival status of LUAD patients, the risk score, and the results of K-M analysis of survival analysis.

To validate the predictive capability of the constructed model, we calculated the risk scores of LUAD patients by using a uniform formula. We examined the expression of ICP-related lncRNAs, survival status scores, and risk scores in LUAD patients in the testing set (Figures 4A–D) and the entire set (Figures 4E–H). In addition, the K-M analysis of the two sets also showed that patients in the low-risk group had a longer OS time than those in the high-risk group (Figures 4D,H *p* = 0.009 and *p* < 0.001).

### Nomogram and independent prognostic factor analysis

**FIGURE 5:**
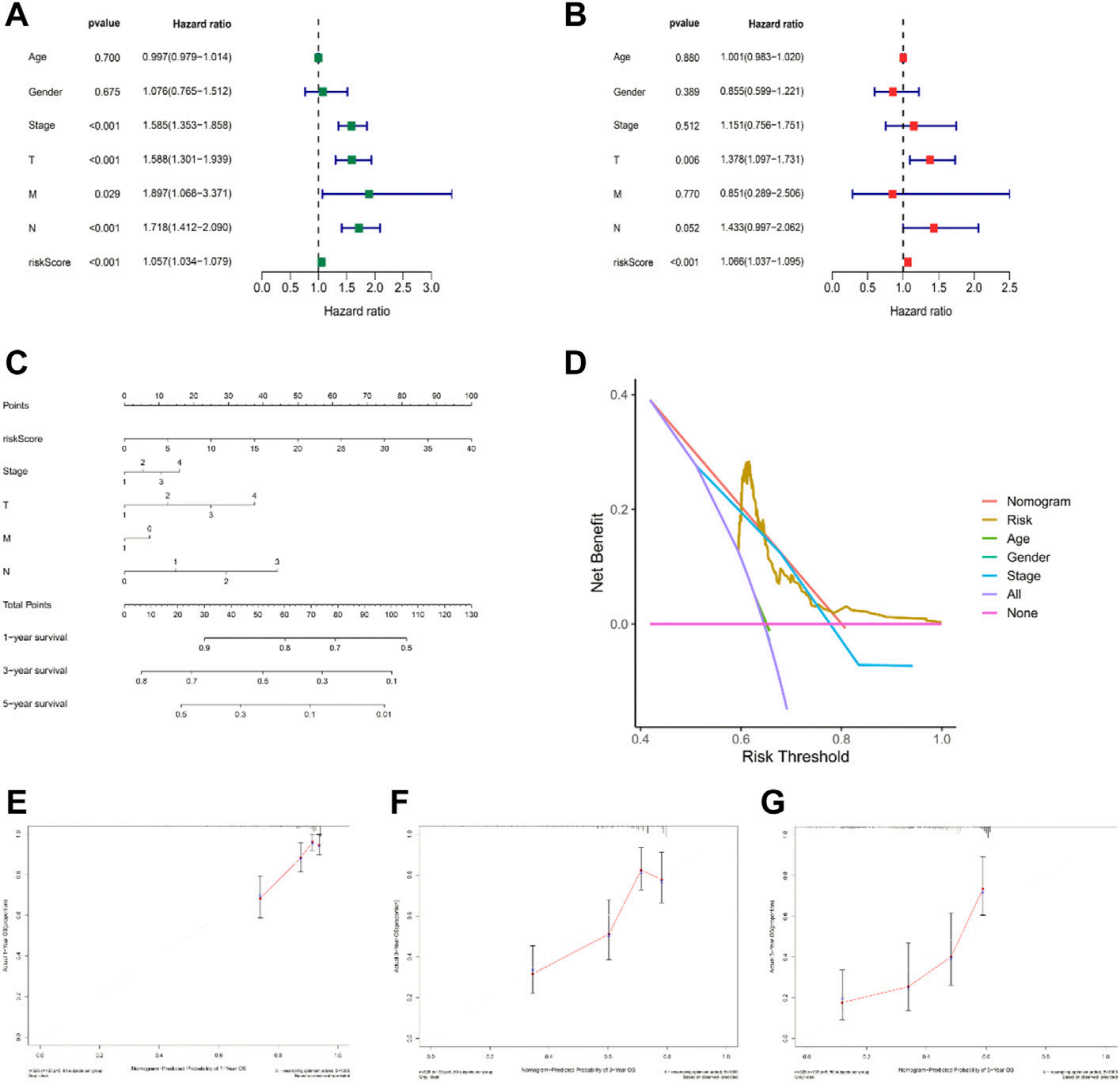
Nomogram and independent prognostic factor analysis. **(A,B)** Result of univariate/multivariate Cox regression analyses. **(C)** Nomogram predicts the probability of the 1-, 3-, and 5-years OS. **(D)** Result of DCA. **(E–G)** 1-, 3-, and 5-years predicted prognosis.

To explore the independent predictive power of risk models and various clinical characteristics for patient outcomes, we performed univariate and all-factor Cox regression analyses, respectively. Univariate Cox regression analysis suggested that age, T/N grade, clinical stage, and risk score were prognostic factors for LUAD patients (Figure 5A, *p* < 0.001), and further multivariate Cox regression analysis showed that the risk score was an independent predictor of prognosis in LUAD patients, the prediction results were reliable, and the confidence level was higher than that of other clinical characteristics (Figure 5B, *p* < 0.001). Therefore, it is reasonable to believe that a risk model based on 14 ICP-related lncRNAs has a significant impact on the survival and prognosis of LUAD patients and were independent prognostic factors. The nomogram (Figure 5C **)** was constructed with a risk score, survival rate, and other clinical features. Then the calibration curve analysis in Figures 5E–G shows the accuracy of the nomogram in predicting the 1-, 3-, and 5-years prognosis of LUAD patients. Furthermore, DCA also indicated that a nomogram has a higher prediction accuracy than a risk model alone (Figure 5D).

**FIGURE 6:**
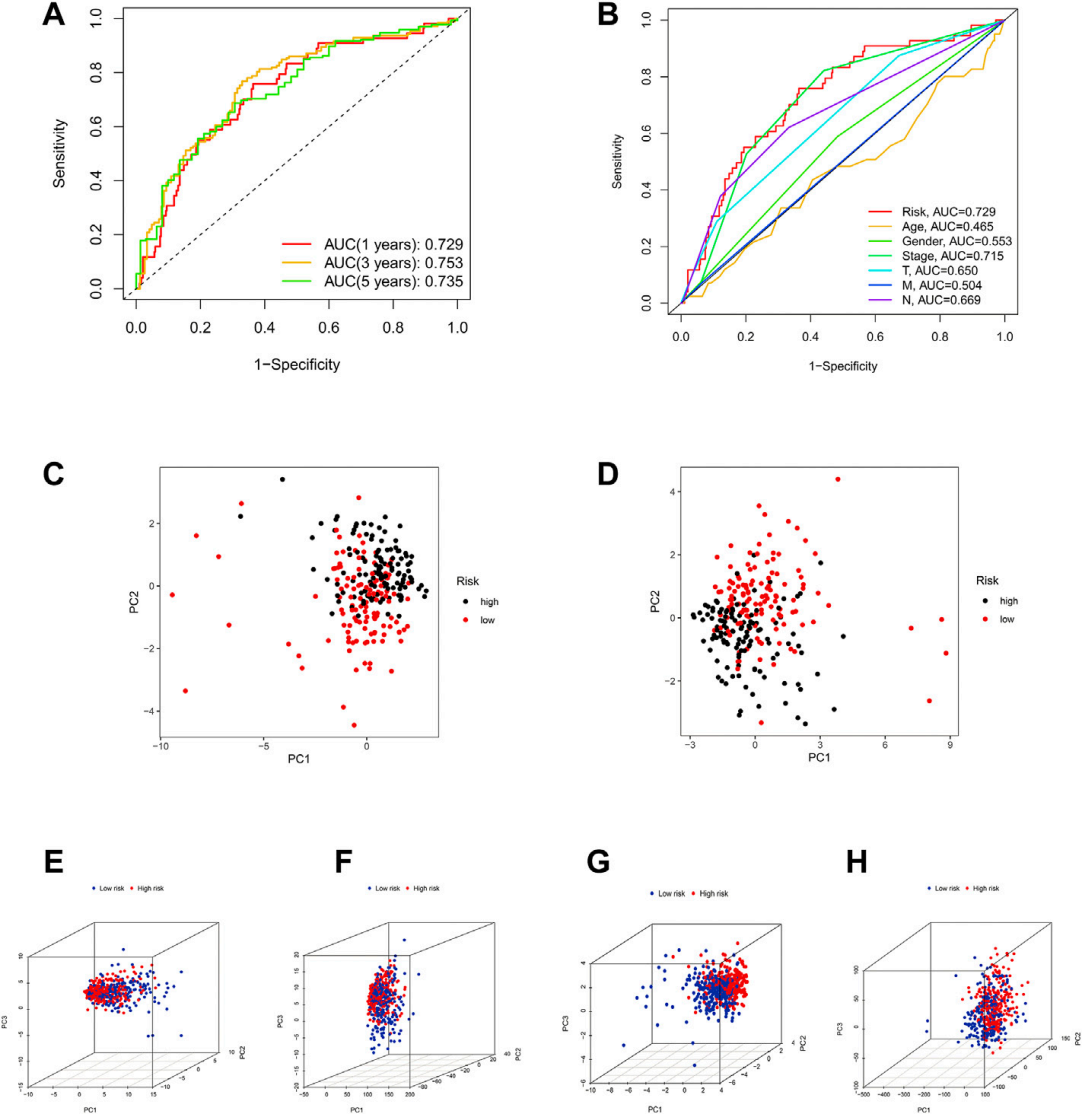
Assessment of the predictive risk model and Principal component analysis. **(A)** The entire set's 1-, 3-, and 5-years ROC curves. **(B)** ROC curves of the clinical characteristics and risk score. **(C,D)** PCA results of testing and training sets. **(E–H)** The PCA result of entire gene expression profiles, ICPDEGs, ICP-related lncRNAs, and risk model according to the 14 hub lncRNAs, respectively.

Otherwise, ROC curve analysis and PCA verify the efficacy of the risk model. The AUC values of the 1-, 3-, and 5-years of OS were 0.729, 0.753, and 0.735, respectively (Figure 6A), which were much higher than other clinical features (Figure 6B). This suggests that these 14 ICP-related lncRNAs are relatively reliable in the prognostic risk model of LUAD. Besides, we applied principal component analysis (PCA) to test the differences between the high-risk and low-risk groups (Figures 6C,D) to further assess the group ability of ICP-related lncRNA models. At the same time, we used PCA to verify the authenticity of the risk model constructed based on the entire gene expression profiles, ICP genes, ICP-related lncRNAs, and risk model according to the 14 hub lncRNAs (Figures 6E–H). The results confirmed that the distributional patterns of the high-risk and low-risk groups were significantly different, which elucidated that the risk model was competent to distinguish the two groups with high accuracy.

### Somatic mutation landscape

**FIGURE 7:**
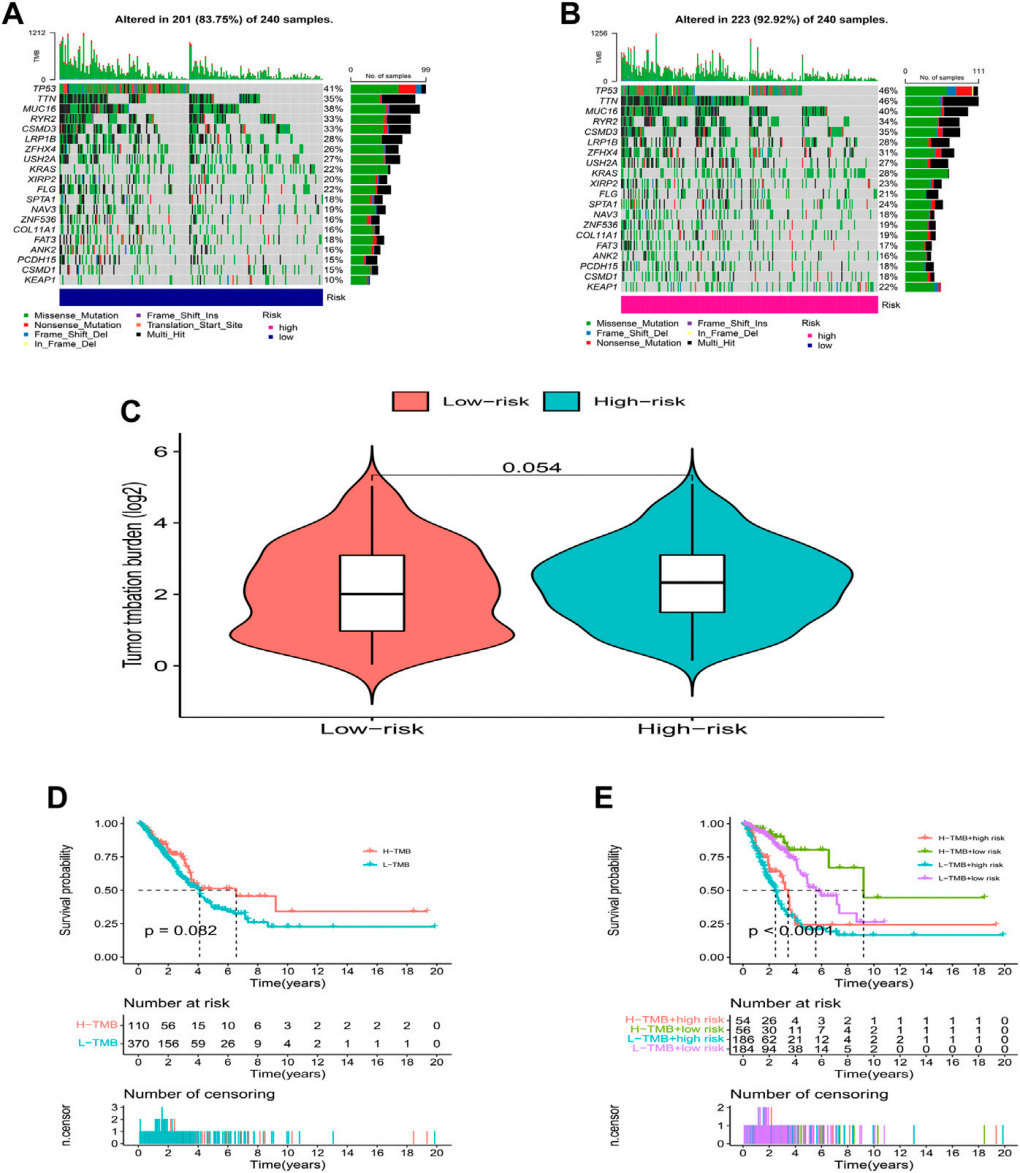
TMB analysis. **(A,B)** The waterfall plot of somatic mutation features established with high- and low-risk groups. **(C)** Tumor mutation burden in the high-risk and low-risk groups. **(D,E)** Kaplan-Meier curve of the OS among the high- and low- TMB groups.

We further analyzed the somatic mutation landscape of LUAD patients. As shown in Figures 7A,B, compared with the low-risk group, the high-risk group showed a higher rate of somatic mutation (92.92% vs. 83.75%), and also had a higher tumor mutational burden (TMB, Figure 7C, *p* = 0.054, with marginal statistical significance). As a classic indicator for evaluating tumor behavior, TMB has been considered reliable in evaluating the prognosis of tumor patients in the past. However, in the survival analysis, we were pleasantly surprised to find that TMB alone could not predict the prognosis of LUAD patients in the high and low TMB groups (Figure 7D, *p* = 0.082), but the combination of the TMB and risk score model can effectively distinguish the prognosis of patients with different risk levels (Figure 7E, *p* < 0.0001).

### Immune signature analysis based on ICP-related lncRNAs

**FIGURE 8:**
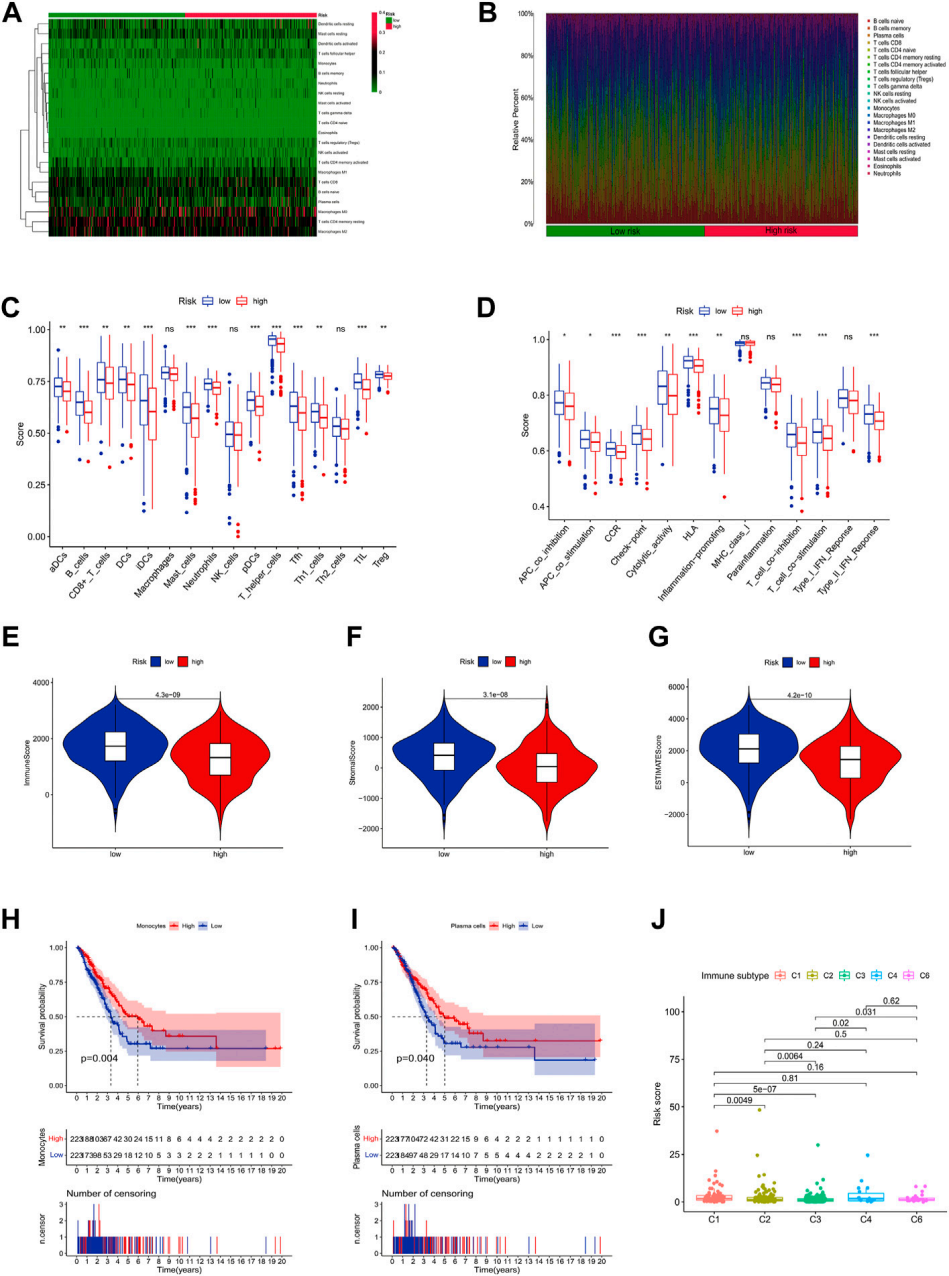
Immune infiltration discrepancy in different risk groups. **(A)** Heatmap of 22 tumor-infiltrating immune cell types in low-risk and high-risk groups. **(B)** Bar chart of the proportions for 22 immune cell types. **(C)** The ssGSEA scores of immune functions in low-risk and high-risk groups. **(D)** Immune cells in low-risk and high-risk groups. **(E–G)** The TME scores between high-risk and low-risk groups. **(H,I)** Survival analysis of combined immune cells. **(J)** Immune subtype.

Figures 8A,B shows the proportion of 22 immune cells in different risk groups in the LUAD samples (Supplementary Table S5). Further ssGSEA immunoassays revealed that a variety of immune cells, including CD8^+^ T cells, and B cells, were less infiltrated in the high-risk group samples, and more diverse in the high-risk group. The immune process activity was also lower than that of the low-risk group (Figures 8C,D). In addition, we found that all three immune scores (stromal score, immune score, and ESTIMATE score) were significantly higher in the low-risk group of LUAD patients, indicating that the TME was different from the high-risk group (Figures 8E–G). The above results suggest that patients at high risk of LUAD were in a more severe immunosuppressed state.

Interestingly, we found that monocytes and plasma cells could well predict the prognosis of patients in different risk groups (Figures 8H,I). Meanwhile, we also found that combined with risk scores, all LUAD samples could be classified into different immune subtypes (Figure 8J), which means that more precise treatment strategies may be adopted for different subtypes in clinical practice.

### Clinical immunotherapy analysis

**FIGURE 9:**
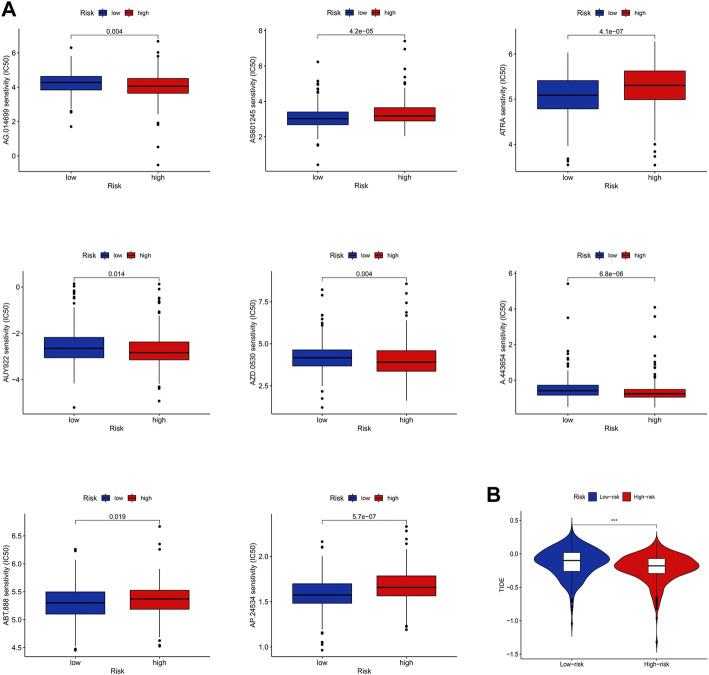
Clinical immunotherapy analysis. **(A)** Results of drug sensitivity analysis. **(B)** Result of TIDE.

For a better clinical therapeutic strategy in LUAD, a drug sensitivity analysis was conducted. The result showed that LUAD patients in the high-risk group had higher IC50s for AS601245, ATRA, ABT.888, and AP.24534, which indicated that these drugs may be clinically less effective for patients in the high-risk group. On the contrary, AG.014699, AUY922, AZD.0530, and A.443654 showed higher IC50 in the low-risk group (Figure 9A). Furthermore, we found that patients in the high-risk group had lower TIDE scores (Figure 9B), which may explain the differences in susceptibility to these drugs.

### Functional enrichment analysis

**FIGURE 10:**
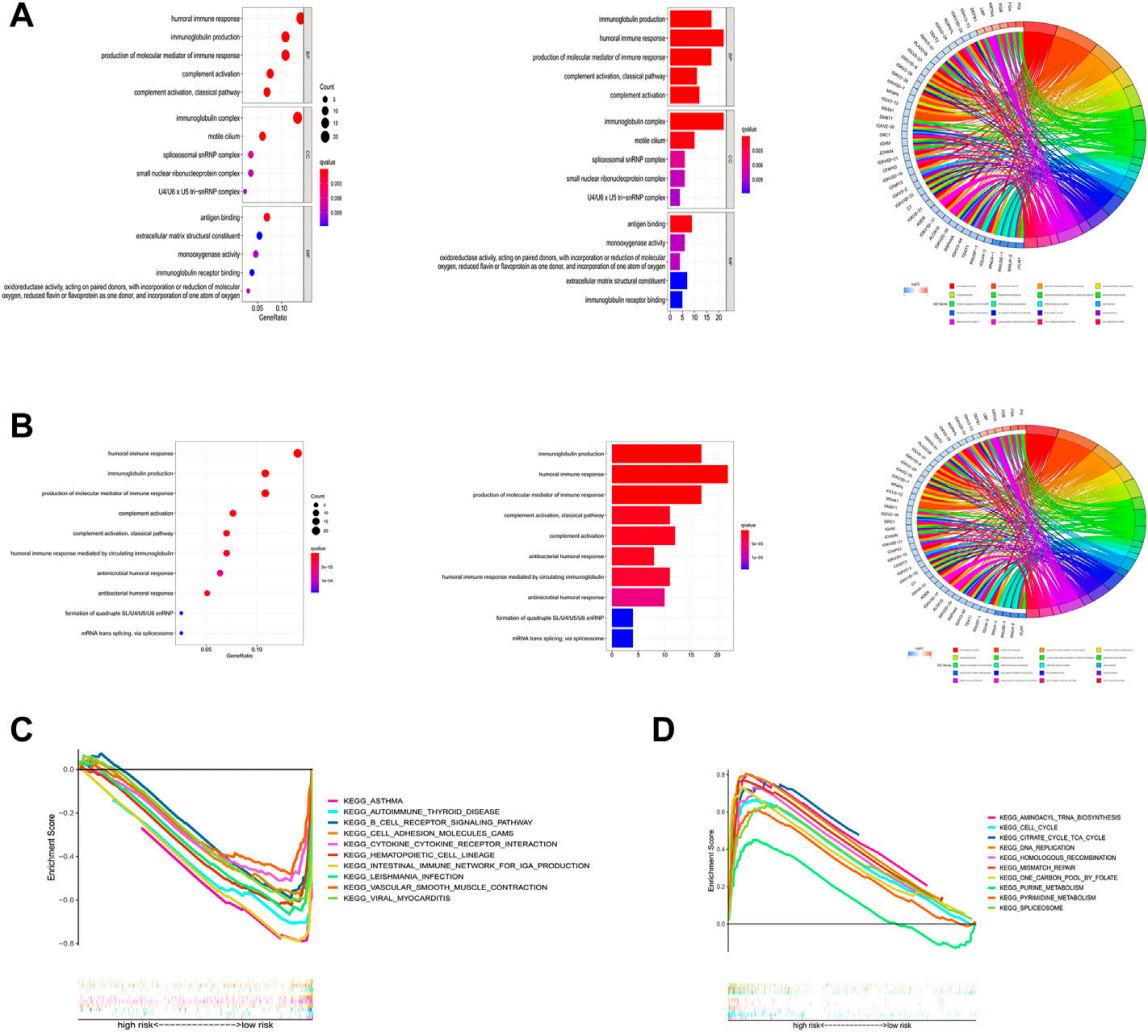
Functional enrichment analysis. **(A)** Result of GO enrichment. **(B)** Result of KEGG enrichment. **(C,D)** Result of GSEA.

To deeply explore how ICP-related lncRNAs produce biological effects, functional enrichment analysis based on differential genes between high and low-risk groups with multi-dimension was performed. The results of the GO analysis suggested that the changes of LAUD involved changes in a variety of immune processes, including humoral immunity and immune complex production (Figure 10A), and the KEGG results also suggested that the disease was highly correlated with humoral immune pathways (Figure 10B). In addition, through GSEA analysis, we found that the B cell receptor pathway and cell adhesion pathway were highly enriched in the low-risk group, while those in the high-risk group were highly correlated with cell cycle and metabolic cycle (Figures 10C,D). These potential mechanisms may point to new directions for the future treatment of LUAD.

## Discussion

Immunotherapy was defined as the use of materials to moderate the function of the immune system to prevent and fight disease (Lizée et al., 2013). It has been widely applied in clinical treatment for cancer like metastatic urothelial carcinoma (Sharma et al., 2016), advanced renal cell carcinoma (Motzer et al., 2015), and other types of cancer. With the growing investigation into immune infiltration, there was gradually reaching a consensus that long non-coding RNAs (lncRNAs) have been associated with cancer immunity regulation and the tumor microenvironment (TME) (Zhou et al., 2021a). Some researchers suggested that immune-related lncRNAs could predict immune cell infiltration and immunotherapy response in patients with liver cancer (Zhou et al., 2021b; Huang et al., 2021), and bladder cancer (Wu et al., 2020), while the association in patients with LUAD is still not understood. At the same time, TME, as a novel hotpot in cancer research, has gained much attention in recent years. Unlike tumor cells, stromal cells also a participant in the initiation, progression, and metastasis of cancer, inducing both beneficial and adverse consequences for tumorigenesis (Stepaniak et al., 1986). Current most advanced TME-directed therapies including antiangiogenic drugs and treatment directed against cancer-associated fibroblasts and the extracellular matrix were already approved or evaluated in trials (Bejarano et al., 2021). Therefore, it is of great importance to investigate the correlation between lncRNAs and immune response in patients with LUAD.

Based on the above characteristics, we conducted a prognostic model aimed at evaluating the association between ICP-related lncRNAs and TMB in patients with LUAD through bioinformatics techniques and survival analysis, providing potential treatment targets for clinical therapy and prognosis.

In this study, we found that 14 ICP-related lncRNAs were significantly associated with LUAD by Cox analysis. Of course, most of them were rarely studied and there were already several investigations about some lncRNAs. Firstly, lncRNA SLC16A1-AS1 has been identified to play a vital role in the metabolic reprogramming as targeting and co-activating of E2F1 in patients with bladder cancer (Logotheti et al., 2020). A study by Tian and Hu (2021) also demonstrated that SLC16A1-AS1 was upregulated in hepatocellular carcinoma and might downregulate miR-141 through methylation to promote cancer cell proliferation. Similarly, in patients with glioblastoma, SLC16A1-AS1 might promote cancer cell proliferation by regulating miR-149 methylation and could be considered a potential diagnostic marker in glioblastoma (Long et al., 2021). Also, there were several studies about the function of SLC16A1-AS1 in oral squamous cell carcinoma (Feng et al., 2020; Li et al., 2022), and triple-negative breast cancer (Jiang et al., 2022). As for lung cancer, Liu et al. (2020) have proved that the expression of SLC16A1-AS1 was significantly lower in NSCLC tissue than that in adjacent tissue, and SLC16A1-AS1 over-expression could block the cell cycle and promote cell apoptosis *in vitro*, suggesting that it might act as a potential biomarker for patients with NSCLC. Then, when it comes to lncRNA AL606489.1, some investigations have proved that it was associated with ferroptosis in LUAD (Guo et al., 2021; Song et al., 2021; Wu et al., 2021) as well as oncosis (Chen et al., 2022), which all demonstrating a relationship between non-apoptotic cell death and LUAD and provide important predictive value for the prognosis of LUAD as well as potential clinical therapeutic targets. Similarly, AC026355.2, a vital immune-associated lncRNA, also showed its prognostic value for identifying immune and necroptosis characteristics in LUAD patients (He et al., 2021; Lu et al., 2022). As for lncRNA AC068792.1, a study by Zhou et al. (2022) proved that this TME-related lncRNA could be acted as a biomarker of clear cell renal carcinoma prognosis and immunotherapy response, while the effect in LUAD still warrants further exploration.

The GO and KEGG enrichment analysis showed that the ICP-related genes were mainly enriched in humoral immune response, immunoglobulin production, and production of the molecular immune response, emphasizing the significance of immune response in cancer development. Then the bioplot showed that the expression of immune cells in the high- and low-risk subgroups mainly focused on plasma cells, monocytes, T cells gamma delta, T cells CD4 memory resting, dendritic cells, and mast cells resting. Subsequent K-M survival analysis demonstrated that the survival probability in plasma cells high-expression subgroup was much higher than that in a low-expression subgroup, illustrating a potential protective value for patients with LUAD. Actually, in a study about the role of tumor-infiltrating B cells and intratumorally-produced antibodies in cancer-immunity interactions, Isaeva et al. (2019) found that plasma cells produced a great number of clonal IgG1, which was not much effective on prognosis, suggesting that IgG1+ tumor-infiltrating B cells might exert a beneficial effect in KRAS mutation cases. While, for the subgroup with higher expression of monocytes, the survival probability also showed the same result as that of plasma cells. As an important component in TME, monocytes were tightly connected with cancer initiation and development. However, an investigation aiming at constructing an immune-related lncRNAs signature in patients with LUAD showed that this signature corrected negatively with B cells, CD4^+^ T cells, and monocytes immune infiltration, and patients with low-risk scores had a higher abundance of immune cells and stromal cells around the tumor (Chen et al., 2021). This contrary result mainly could be explained that the function of tumor-associated monocyte/macrophage lineage cells (MMLCs) might be different in human tumors, especially in the early stages of the disease (Singhal et al., 2019). Classical “inflammatory” monocytes promote tumor growth and metastasis, however, nonclassical “patrolling” monocytes contribute to cancer immunosurveillance and may be targeted for cancer immunotherapy (Qian et al., 2011; Hanna et al., 2015). Thus, further studies are warranted to explore specific mechanisms in patients with LUAD.

Then we analyzed the immune score between high-risk and low-risk subgroups and found that significant differences were shown in tumor-infiltrating lymphocyte (TIL), cytolytic activity, and major histocompatibility complex. Indeed, the efficacy of clinical immunotherapy varies and depends on the amount and properties of TILs, and in general, TILs represent a favorable prognostic factor in NSCLC (Guo et al., 2018; Gueguen et al., 2021). Federico et al. (2022) demonstrated that though the number of infiltrating T cells was not associated with patient survival, the nature of the infiltrating T cells could have a prognostic value in NSCLC and became potential therapeutic approaches for clinical care. As for HLA, Datar et al. (2021) claimed that patients with cancer cell-selective HLA-B, HL-C or HLA class-II downregulation displayed decreased T cells and NK-cell infiltration, then associated with shorter overall survival, which broaden a novel insight into clinical therapeutic targets. While, for advanced NSCLC treated with immune checkpoint blockade, HLA class-I genotype was not correlated with survival, which emphasizes the correlation between immune checkpoints and HLA (Negrao et al., 2019). Further studies are needed to claim deeper relationships and provide novel insights.

In addition, there are still some limitations in our study. First, our current study was limited to the bioinformatics level and no external experiments were conducted to validate the results. Second, although we validated the model by constructing a valid prediction model, the model construction relied only on the TCGA database, which could potentially lead to less credible study results.

In conclusion, to explore the connection between lncRNAs and immune infiltration in patients with LUAD, we conduct a relatively overall and comprehensive prognostic model to evaluate the expression of various immune cells and survival probability through bioinformatics techniques, confirming that immune response played a vital role in the progression of cancer and the crosslink between immune infiltration and lncRNAs, which could provide potential therapeutic targets for clinical care.

## Data Availability

The datasets presented in this study can be found in online repositories. The names of the repository/repositories and accession number(s) can be found in the article/Supplementary Material.

## Supplementary material

SUPPLEMENTARY TABLE S1 ICP-associated genes.

SUPPLEMENTARY TABLE S2 Clinical information.

SUPPLEMENTARY TABLE S3 Univariate COX regression analysis.

SUPPLEMENTARY TABLE S4 Multivariate COX regression analysis.

SUPPLEMENTARY TABLE S5 The proportion of 22 immune cells in different risk groups.

## Abbreviations

CAFs, cancer-associated fibroblasts; CAR, chimeric antigen receptor; CNV, copy number variation; CTLA-4, cytotoxic T lymphocyte antigen-4; GDSC, genomics of drug sensitivity in cancer; ICP, immune checkpoints; ICPDEGs, immune checkpoint genes; IL-2, interleukin 2; lncRNAs, long non-coding RNAs; LUAD, lung adenocarcinoma; MDSCs, myeloid-derived macrophages; NSCLC, non-small cell lung cancer; OS, overall survival; PD-1, programmed cell death protein 1; SCLC, small cell lung cancer; TIDE, tumor immune dysfunction and exclusion; TIL, tumor-infiltrating lymphocyte; TMB, tumor mutational burden; TME, tumor microenvironment; Tregs, regulatory T cells.
